# Research on In-Plane Thermal Conductivity Detection of Fuel Cell Bipolar Plates Based on Laser Thermography

**DOI:** 10.3390/s24134206

**Published:** 2024-06-28

**Authors:** Yang Li, Dexin Hou, Feng Li, Lianghui Huang, Zhihua Huang, Yuehuan Zhang, Yongping Zheng, Leipeng Song, Bingqiang Huang, Zhengshun Fei, Xinjian Xiang

**Affiliations:** 1 School of Automation and Electrical Engineering, Zhejiang University of Science and Technology, Hangzhou 310023, China; 120048@zust.edu.cn (Y.L.); 120036@zust.edu.cn (L.H.); 108019@zust.edu.cn (Y.Z.); 124018@zust.edu.cn (L.S.); 114023@zust.edu.cn (B.H.); zsfei@zust.edu.cn (Z.F.); 2 College of Metrology Measurement and Instrument, China Jiliang University, Hangzhou 310018, China; dexin_hou@163.com; 3 Hangzhou Youchuan Technology Co., Ltd., Hangzhou 310018, China; 4 Yongkang Valid Technology Co., Ltd., Jinhua 321300, China; lifeng@validtools.com (F.L.); gxylzhihua@163.com (Z.H.); 5 Hangzhou Yodosmart Automotive Technology Co., Ltd., Hangzhou 311100, China; yuehuan_zhang@126.com

**Keywords:** nondestructive testing, infrared thermographic testing, thermal conductivity, graphite bipolar plates

## Abstract

The thermal properties of bipolar plates, being key elements of polymer electrolyte membrane fuel cells, significantly affect their heat conduction and management. This study employed an innovative approach known as a heat flow loop integral method to experimentally assess the in-plane thermal conductivity of graphite bipolar plates, addressing the constraints of traditional methods that have strict demands for thermal stimulation, boundary or initial conditions, and sample size. This method employs infrared thermal imaging to gather information from the surface temperature field of the sample, which is induced by laser stimulation. An enclosed test loop on the infrared image of the sample’s surface, situated between the heat source and the sample’s boundary, is utilized to calculate the in-plane heat flow density by integrating the temperature at the sampling locations on the loop and the in-plane thermal conductivity can be determined based on Fourier’s law of heat conduction. The numerical simulation analysis of the graphite models and the experimental tests with aluminum have confirmed the precision and practicality of this method. The results of 1060 aluminum and 6061 aluminum samples, each 1 and 2 mm in thickness, show a deviation between the reference and actual measurements of the in-plane thermal conductivity within 4.3% and repeatability within 2.7%. Using the loop integral method, the in-plane thermal conductivities of three graphite bipolar plates with thicknesses of 0.5 mm, 1 mm, and 1.5 mm were tested, resulting in 311.98 W(m·K)^−1^, 314.41 W(m·K)^−1^, and 323.48 W(m·K)^−1^, with repeatabilities of 0.9%, 3.0%, and 2.0%, respectively. A comparison with the reference value from the simulation model for graphite bipolar plates with the same thickness showed a deviation of 4.7%. The test results for three different thicknesses of graphite bipolar plates show a repeatability of 2.6%, indicating the high consistency and reliability of this measurement method. Consequently, as a supplement to existing technology, this method can achieve a rapid and nondestructive measurement of materials such as graphite bipolar plates’ in-plane thermal conductivity.

## 1. Introduction

Polymer electrolyte membrane (PEM) fuel cells are designed with a layered structure of single cells or membrane electrode assemblies (MEAs) to cater to the distinct power generation requirements of various applications. These PEM fuel cells or MEAs are arranged in a stack with bipolar plates (BPPs) inserted between them for separation. Currently, PEM fuel cells are increasingly recognized as viable energy sources and potential eco-friendly energy conversion tools for various portable uses [1]. The uses encompass e-bikes, drones, and forklifts, among others [2].

Since PEM fuel cells typically operate at temperatures below 100 °C, excessive heat generated during their operation can cause the temperature inside the cell to rise, further reducing the water content in the electrolyte membrane of fuel cells. In severe cases, this could cause the polymer electrolyte membrane to dehydrate, significantly reducing ion conductivity. As a result, a deceleration in the rate of electrochemical reactions may adversely affect the cell’s performance and longevity. Consequently, effective thermal management is crucial for enhancing the reaction effectiveness, durability, and general performance of PEM fuel cells [3]. Maintaining adequate thermal management is critical for the continued advancement and successful commercialization of PEM fuel cell technology [4].

The distribution of temperature within a cell is controlled by heat transfer across its different components, including bipolar plates, gas diffusion layers, catalyst layers, and the electrolyte membrane [3]. One of the primary functions of bipolar plates, as a crucial element in fuel cell stacks, is to facilitate uniform heat distribution, efficient heat transfer, and effective cooling, thus maintaining optimal operating temperatures and contributing to the overall thermal management of the fuel cell system. Therefore, in the fuel cell design stage, the main focus is on pinpointing the ideal material for bipolar plates and related thermal management strategies [2]. This is crucial for optimizing the performance and efficiency of the fuel cell system.

Understanding the thermal properties of membrane-electrode components, particularly bipolar plates, is vital for efficient thermal management in fuel cells. Tang’s research focused on enhancing the thermal conductivity of bipolar plates to optimize thermal management in PEM fuel cells. The suggestion was made to employ a direct heat dissipation method instead of liquid cooling. This involves enhancing the thermal conductivity of the bipolar plates, simplifying the flow channels to reduce costs, and achieving similar temperature differences as circulating liquids, thereby improving thermal management effectiveness [5]. Additionally, precise prediction of temperature distribution is crucial for enhancing the thermal management of fuel cells. A variety of modeling methods have been suggested to predict the temperature distribution in PEM fuel cells under various operating conditions. Precise prediction of temperature distribution within a fuel cell depends on the essential analysis of its thermal properties, which includes the varied thermal conductivities of its components, significantly aiding modelers in their estimations [6]. However, the absence of detailed data in the literature about the varied thermal conductivity of fuel cell elements presents a hurdle for modelers. Achieving precise temperature distribution estimation becomes challenging without an in-depth knowledge of the thermal conductivity of cell components.

Numerous investigations have been conducted by different researchers to assess the thermal conductivity of fuel cell elements, such as gas diffusion layers (GDLs) [7,8], microporous layers (MPLs) [9,10,11], polymer electrolyte membranes (PEMs) [12], etc., and thermal conductivity variations under different compaction pressures were measured for some fuel cell components. At present, there are many experimental methods to measure thermal properties [13,14,15,16,17,18,19], which further demonstrate the relevance and importance of thermal properties detection in thermal analysis and materials science. However, to the author’s knowledge, there is no standard experimental method to characterize the in-plane thermal conductivity of bipolar plates, and only a few experimental assessments of the thermal conductivity of bipolar plates in sufficient detail have been carried out. A custom-made thermal contact resistance (TCR) machine, designed based on the ASTM Standard C-177’s guarded heat flux meter device principle [20], was utilized to evaluate the thermal conductivity of graphite bipolar plates in Ref. [21]. To accurately measure the thermal conductivity, it is essential to determine the one-dimensional heat flow through the sample and measure the temperature drop across the sample with precision. Therefore, this detection principle determines the measurement of the through-plane thermal conductivity of the bipolar plate. The material’s thermal conductivity shows considerable variation between through-plane and in-plane directions, owing to the anisotropic microstructure of most graphite bipolar plates. Compared with the through-plane direction, the in-plane direction demonstrates significantly greater thermal conductivity. The laser-flash technique involves heating a thin circular sample’s surface with a specific thickness using a uniform laser pulse, followed by measuring the temperature response on the reverse side to determine thermal diffusivity, thereby indirectly obtaining thermal conductivity. Employing the in-plane type of holders is essential because of the anisotropic characteristics of the samples [22] and the heat transfer in the in-plane directions of a sample by the controlling functions of masks and sample holders, enabling the measurement of the in-plane thermal conductivity of the sample [7]. Additionally, to minimize the impact of the sample surface’s roughness on measuring thickness, it is essential to use highly accurate surface grinding techniques [23], and the sample thickness needs to be sufficiently thin to satisfy the conditions of two-dimensional heat conduction, such as various film materials with thicknesses on the micron scale [24,25,26]. In addition, for the flash method, thermal conductivity can only be determined from previous knowledge of thermal capacity and density.

In the limited modeling studies that consider anisotropy, parametric investigations show that adding anisotropic properties has a significant impact on the distribution of current density and the relative significance of the limiting transport process. Therefore, accurately determining the in-plane thermal conductivity serves as a crucial parameter for the thermal analysis and management of PEM fuel cells and stacks [27].

In addition, in order to ensure the accuracy of measurement results, traditional methods require more sophisticated experimental equipment, pre-treatments, and operational procedures to strictly control experimental conditions, including thermal excitation, boundary conditions, and overall material size. For example, prior to taking measurements with a guarded heat flow meter device, some surface analytical tests are performed on each sample using a surface dial indicator to ensure acceptable surface flatness [21].

Presently, detection techniques mainly concentrate on assessing the through-plane thermal conductivity of bipolar plates, with graphite bipolar plate blank samples [21] or graphite bare materials being the subjects of testing. Limited studies have been conducted on the in-plane thermal conductivity of bipolar plate products. Furthermore, the industry predominantly depends on data from bare materials acquired via the guarded heat flow method as a standard indicator. Yet, fuel cell users focus more on the completed bipolar plate products than on the bare materials. Employing bare material testing data hinders the precise analysis of thermal design and management. Consequently, an uncomplicated experimental approach is essential for directly and precisely assessing the in-plane thermal conductivity of bipolar plate products.

To achieve a rapid and nondestructive test of the in-plane thermal conductivity of bipolar plate products, an innovative experimental technique utilizing infrared thermography is suggested based on early research [28]. The method relies on identifying temperature field information resulting from laser heating on a bipolar plate surface in infrared imagery. Once a stable temperature field is formed, the in-plane thermal conductivity of the tested sample can be calculated by measuring the heat flow passing through the sample and the temperature gradient formed on the specified test loop, combined with Fourier’s law. This method, unlike conventional transient methods, does not require rigorous management of boundary and initial conditions. Therefore, there is no need for preliminary treatment, including the processing of the material under test, the application of particular excitation modes, thermal insulation measurements, and other specific experimental conditions. As a consequence, the experimental process is simplified, and the requirements for sample characteristics and processing are reduced.

The key parameters determined for bipolar plates provide important input parameters for the mathematical modeling of fuel cells. So, this research is expected to address the missing values of the in-plane thermal conductivity in bipolar plate product studies and assist modelers in assessing the temperature distribution in an operating PEM fuel cell.

## 2. Heat Conduction Model of Materials

### 2.1. Theoretical Analysis of Measurement Model of In-Plane Thermal Conductivity

**Figure 1:**
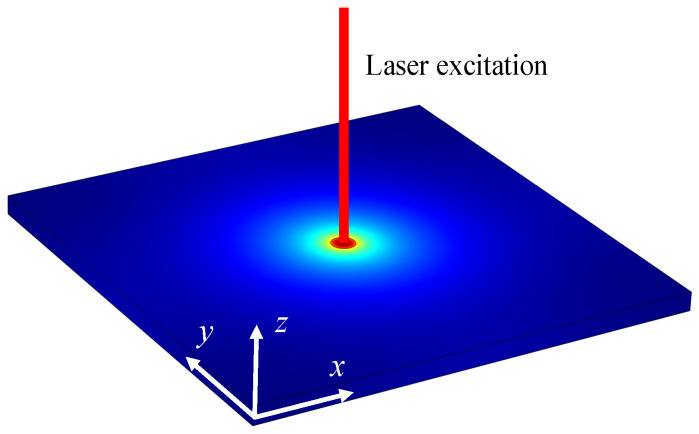
Schematic for heat conduction of a material in the in-plane direction.

Exposing a material’s surface to a steady point heat source, such as from a laser, results in the transfer and diffusion of heat flow from this point to the entire material via heat conduction. At the same time, heat is transferred between the sample and the surrounding environment by convection and radiation. Figure 1 illustrates the heat conduction model.

The proposed model is predicated on these hypotheses:

(1) The in-plane thermal conductivity of the material, surface heat transfer coefficient (encompassing convection and radiation), and the cross-sectional areas for thermal conduction remain unchanged.
(2) The surface convection thermal resistance significantly surpasses the normal surface resistance, and the material’s temperature is deemed consistent across the section at a certain heat transfer distance from the point heat source.

Additionally, in the case of flat samples with very small thicknesses, the temperature gradient along the through-plane direction is negligible compared with the in-plane direction. The smaller the sample thickness, the smaller the approximate introduced error. Consequently, the study and measurement concentrate on the in-plane thermal conductivity of the sample.

The heat, *q_heat_*, generated by laser excitation is transferred through the following two paths:

(1) The heat transferred in the in-plane direction of the sample is denoted as *q_in_*;
(2) The heat loss caused by the exchange of heat between the surface of the sample and the surrounding environment is denoted as *q_surf_*.

According to the law of energy conservation, the heat transfer is balanced in thermal equilibrium and can be expressed as:

*q_heat_* = *q_in_* + *q_surf_* (1)

The expression of Fourier’s law of heat conduction is as follows:

(2) $$\Phi =-\lambda \cdot A\frac{dT}{dx}$$

where *T* is the temperature; *x* is the space coordinates of the material; *Φ* is the heat flow through a specific cross-sectional area per unit of time; *λ* is the in-plane thermal conductivity of the sample; *A* is the cross-sectional area through which heat is transferred; *dT/dx* is the temperature gradient along the direction of heat flow.

**Figure 2:**
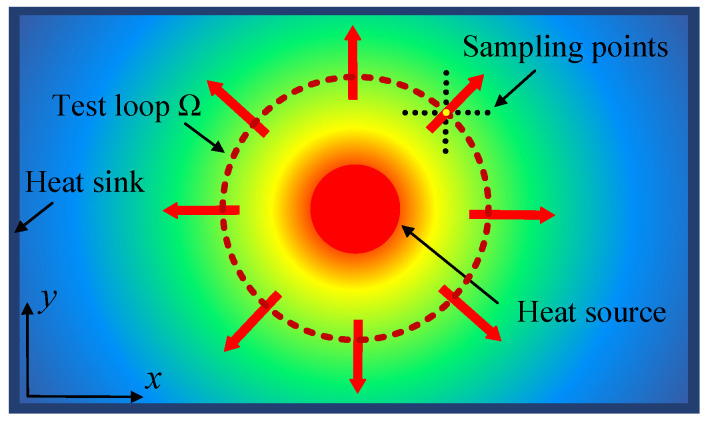
Surface temperature distribution of the sample under laser excitation.

The sample is continuously heated using a laser with constant power as the heat source. Once the heat conduction in the sample reaches thermal equilibrium in the plane direction, the temperature distribution *T*(*x*, *y*) on the sample surface is observed and recorded using an infrared thermal camera, as illustrated in Figure 2.

A closed test loop Ω on the surface of the sample was chosen that lies between the heat source and the sample boundary (i.e., heat-sink), and a closed region that fully surrounds the heat source was constructed, as shown by the dashed line in Figure 2. A point on the test loop as the central sampling point was selected. From this point, a series of sampling points were set along the X- and *Y*-axis. The temperature gradients *GradT_x_* and *GradT_y_* along the two orthogonal directions were obtained by least-squares fitting of the sampling points. The expression of the fitting function is as follows:

(3) $$f(T_{x})={GradT}_{x}\cdot T_{x}+P_{x}$$

(4) $$f(T_{y})={GradT}_{y}\cdot T_{y}+P_{y}$$

where *T_x_* and *T_y_* are the temperature values of sampling points along the X- and *Y*-axis, respectively, and *P_x_* and *P_y_* are the parameters obtained from the fitting.

Based on these two temperature gradients, the normal directional component of the central sampling point can be obtained, which is the outward vector *GradT*_(*x*,*y*)_ from the heat source to the central sampling point, expressed in complex form as shown below.

(5) $${GradT}_{\left(x,y\right)}={GradT}_{x}+i\cdot {GradT}_{y}$$

The normal temperature gradients at each sampling point on the test loop are calculated and accumulated to obtain the integral value, as shown below. This helps reduce the impact of spatial resolution and temperature noise in infrared thermography.

(6) $$\oint_{\Omega }\frac{\partial T(x,y)}{\partial n(x,y)}\cdot dl=\sum_{\Omega }{GradT}_{\left(x,y\right)}$$

Multiplying the integral result by the thickness of the sample yields the product of the temperature gradient in the direction of heat transfer and the heat transfer surface area within the sample, which can be expressed as:

(7) $$D\cdot \oint_{\Omega }\frac{\partial T(x,y)}{\partial n(x,y)}\cdot dl=\frac{dT}{dx}\cdot A$$

where ∂T(x,y)/∂n(x,y) is the directional derivative of the sample surface temperature field at each sampling point along the normal direction outside the test loop; *D* is the thickness of the sample. Combining the above formula, we can obtain the in-plane thermal conductivity *λ* of the test sample, which is given by:

(8) $$\lambda =\frac{q_{in}}{D\cdot \oint_{\Omega }\frac{\partial T(x,y)}{\partial n(x,y)}\cdot dl}=\frac{q_{heat}-q_{surf}}{D\cdot \oint_{\Omega }\frac{\partial T(x,y)}{\partial n(x,y)}\cdot dl}=\frac{q_{heat}-q_{surf}}{D\cdot \sum_{\Omega }{GradT}_{\left(x,y\right)}}$$

The heat flow loop integration method integrates the heat flow density on the heat transfer section and utilizes the continuity of total power to calculate the in-plane thermal conductivity. This avoids the impact of non-uniform excitation on the test results and reduces the requirements for the heat source compared with traditional thermal property measurement methods, such as the step-wise transient (SWT) method [29,30] and the periodic heat flow (PHF) method [31].

### 2.2. Estimation of Heat Loss and Analysis of Loop Radius Selection

Based on the aforementioned Equation (8), the accuracy of in-plane thermal conductivity measurement relies on the accurate estimation of surface heat loss *q_surf_*. When the air pressure and humidity remain constant, there are no airflow disturbances, and the surface temperature distribution of the sample remains unchanged. The surface heat loss of the sample is mainly determined by the ambient temperature and the surface heat transfer coefficient (which includes convection and radiation).

When the sample reaches thermal equilibrium, the temperature difference between the surface of the test loop and the ambient temperature Tenv is expressed as follows:

(9) $$\Delta T=T(x,y)-T_{env}$$

According to Newton’s cooling formula, the surface heat loss of the sample can be expressed as follows:

(10) $$q_{surf}=\sum_{(x,y)\in \Omega }\left({hS}_{\mathrm{loop}}\Delta T\right)$$

where *S*_loop_ is the area inside the test loop taken on the sample, and *h* is the surface heat transfer coefficient of the combined heat transfer, including the convection and radiation heat transfer coefficients. These coefficients depend on various factors, such as shape, roughness, inclination, and physical properties of the solid surface, as well as the fluid state, surface emissivity, and temperature difference between the solid surface and the fluid. This is the factor that affects the surface combined heat transfer coefficient, which is why the empirical value of this coefficient is usually estimated on the basis of empirical values or empirical formulas and experimental conditions. Subsequently, the surface heat loss *q*_surf_ can be calculated using Equation (10), and the in-plane thermal conductivity *λ* can be calculated by substituting the above estimated parameters into Equation (8).

In the analysis of a sample’s surface temperature field data, choosing a suitable loop radius for determining the integral of the normal temperature gradient at test loop sampling points is crucial. On the basis of the above theoretical model, a test loop between a heat source and a boundary heat sink can be established. However, for thicker samples, the closer to the laser area, the greater the temperature gradient in the through-plane direction, and thus, the larger the error caused by through-plane heat conduction. On the other hand, in order to avoid excessive temperature fluctuations at the boundaries of the heating area due to uneven heating by the laser, which may significantly affect the test results, it is recommended to set a larger radius for the test loop to reduce the above impact. However, the large test loop means the temperature readings at the sampling point have a low signal-to-noise ratio, as heat losses on the surface of the sample increase with the dissipation area within the loop. At the same time, the temperature at the sampling points drops, which can also affect test results. Therefore, it is necessary to set the loop radius within an appropriate range to balance the accuracy and repeatability of the experimental method.

### 2.3. Finite Element Simulation Analysis and Model Verification

To verify the correctness of this model, the point heat source excitation and heat conduction of a sample was simulated using the COMSOL Multiphysics 5.4 software. The corresponding finite element (FE) model of a graphite sample and a graphite bipolar plate with dimensions of 100 mm × 100 mm × 1 mm was constructed, and heat transfer data were calculated with this FE model. The channel dimensions of the graphite bipolar plate model are 80 mm × 1 mm × 0.4 mm, with a total of 35 channels. The corresponding thermophysical parameters of the two models are listed in Table 1. The laser excitation on the surface of the sample is simulated by a circular area with a 5 mm diameter and a heat flow of 0.39 W/mm^2^. The initial temperature was 294.15 K, and the boundary condition of the model was convection heat transfer with a coefficient of *h* = 10 W/(m^2^·K). The duration of the point heat source was assumed to be 50 s, with a time step of 0.1 s. The radius from the loop to the edge of the laser area is 14 × 10^−3^ m.

**Figure 3:**
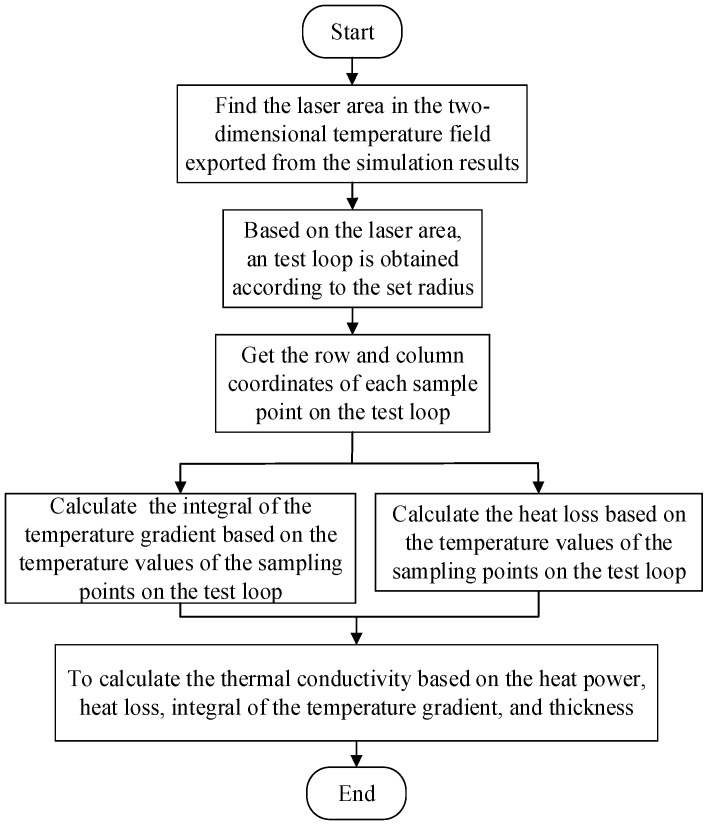
Thermal conductivity calculation flowchart via loop integral method.

**Figure 4:**
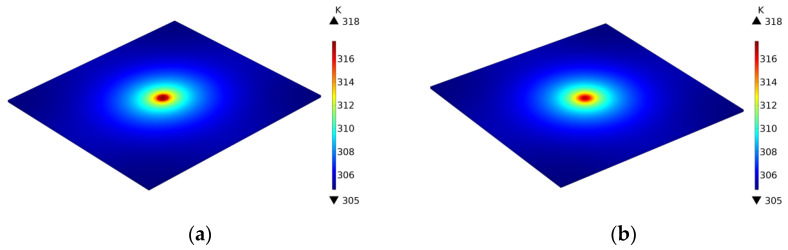
Thermal imaging of graphite material simulation model (**a**) Laser excitation surface. (**b**) Back surface.

**Figure 5:**
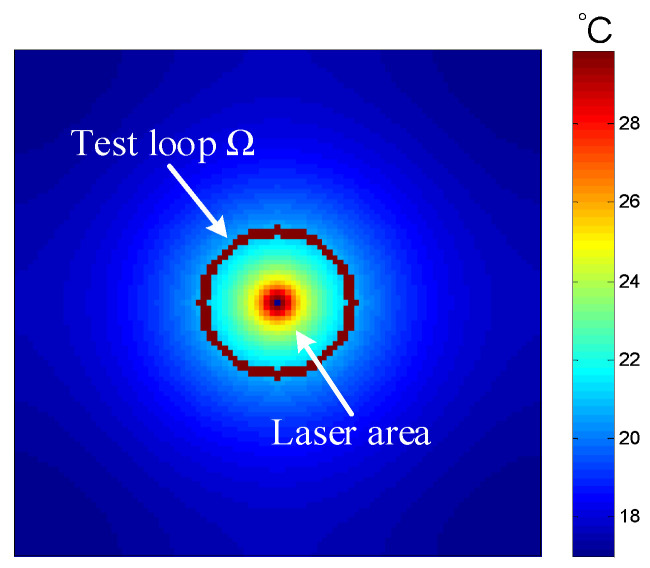
Schematic diagram of laser and test loop for graphite material.

**Figure 6:**
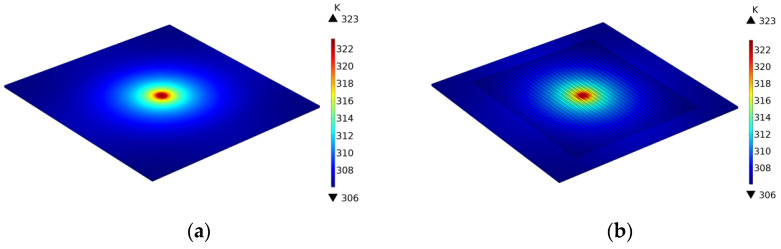
Thermal imaging of graphite bipolar plate simulation model (**a**) Laser excitation surface. (**b**) Back surface.

**Figure 7:**
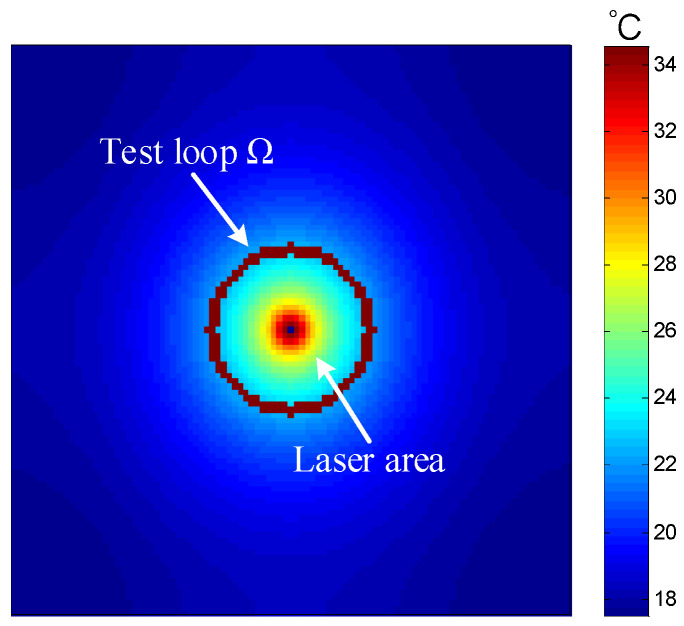
Schematic diagram of laser and test loop for graphite bipolar plate.

The process of calculating the thermal conductivity of a simulation model using the loop integral method is shown in Figure 3. Taking the thermal conductivity value entered into the model as a reference value, the results show, as indicated in Table 2, that the calculated in-plane thermal conductivity of both models deviates from the reference values by less than 2%. This demonstrates the correctness of the heat conduction model. (See Figure 4, Figure 5, Figure 6 and Figure 7).

## 3. Experimental Evaluation of In-Plane Thermal Conductivity of Sample

### 3.1. Experimental Schemes

**Figure 8:**
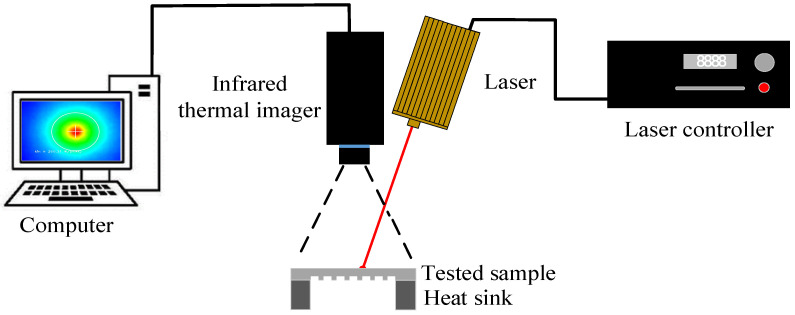
Diagram of the experimental device.

The schematic of the test apparatus principle is shown in Figure 8. The tested sample is mounted on a clamping support, and the sample’s excited surface temperature rapidly increases when the laser emits a high-energy beam. Thereafter, the thermal image of the excited surface is detected by an infrared thermal imager, which provides the temperature distribution during the heat diffusion. In this device, the thermal camera is positioned directly above the test sample, and a custom-made mechanical platform is used to ensure that the thermal camera is positioned vertically, allowing for direct observation of the sample below. The detection time was 30 s, and the ambient temperature during the experiment was 20 ± 0.5 °C. The thermal conductivities of the samples were measured using the described model.

### 3.2. Experimental Apparatus

**Figure 9:**
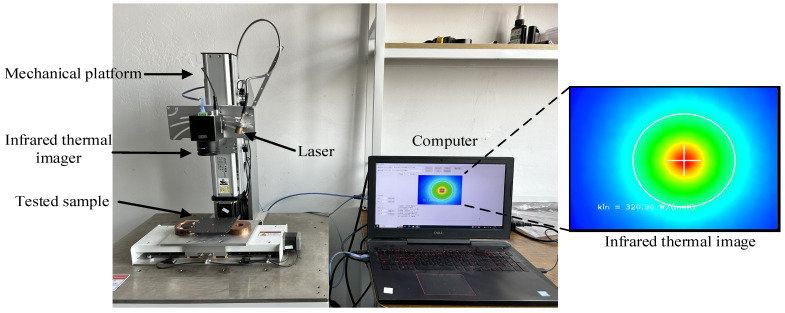
Experimental test on the in-plane thermal conductivity of bipolar plate.

The experiment employed a laser (RAL915AIO4-10.0W) manufactured by Beijing RaySource Technology Co., Ltd., (Beijing, China) to generate an infrared beam with wavelength of 1064 nm ± 1 nm, output power continuously adjusted between 0 to 10 W. An infrared thermal imager (MAGNITY MAG32) with a space resolution of 384 × 288 pixels, temperature measurement accuracy of ±2 °C, and frame rate of 50 Hz is used for temperature measurement. The other main parameters of the apparatus are listed in Table 3 and Table 4. The infrared thermal images were displayed on a computer. The overall look of the experimental setup and an infrared thermal image acquired in an experimental test are shown in Figure 9.

## 4. Measurement Results and Data Analysis

### 4.1. Analysis of Experimental Results of Aluminum Materials

In order to confirm the practicality and consistency of the heat flow loop integral technique, experiments were initially performed on isotropic materials with established thermal conductivity, namely 1060 aluminum and 6061 aluminum. The reference values of thermal conductivity for the two aluminum materials are 236 W/(m·K) and 169 W/(m·K), respectively, derived from ’Heat transfer’ [32]. To enhance laser energy absorption, minimize ambient radiation impact, and prevent the laser beam light from reflecting off the detector, all sample surfaces were uniformly sprayed with black matte paint. The layer was adequately thin (approximately 20 to 60 μm) to prevent negative impacts on the in-plane thermal properties measurements of the material. The samples exhibited a surface emissivity of 0.95 ± 0.1. This value was derived by comparing the temperature measurements of a contact temperature detector (a thermocouple) with the radiation–temperature measurement of the IR detector. The radius from the loop to the edge of the laser area is 14 mm. Moreover, in experiments relying on natural convection, the empirical value of the surface heat transfer coefficient can be taken as *h* = 10 W/(m^2^·K).

**Figure 10:**
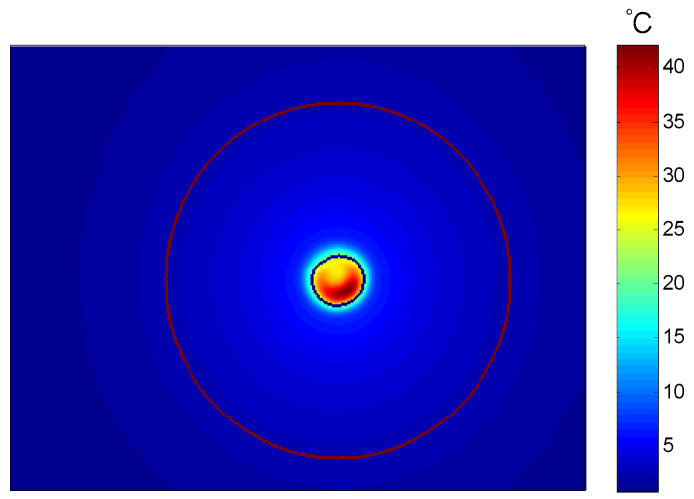
Infrared thermal image of aluminum material.

The average values of the in-plane thermal conductivity of the two aluminum materials with dimensions of 100 mm × 100 mm × 1 mm were obtained from ten independent measurements. The results are listed in Table 5 and Table 6. Subsequently, the 1060 aluminum samples with thicknesses of 1 mm and 2 mm were also tested under the same experimental conditions, and the results are listed in Table 6 and Table 7. An infrared thermal image of the aluminum material is shown in Figure 10.

Results showed that the deviation of three samples from the reference and measured values was within 4.3%. In addition, the absolute and relative standard deviations for ten measurements were calculated and considered as type A uncertainty [33]. These values are reported in Table 5, Table 6 and Table 7. The repeatability was found to be within 2.7%. Therefore, the feasibility and accuracy of the measurement method were confirmed with experimental measurements. For the 1060 aluminum material, the measured values of samples with a 2 mm thickness and 1 mm thickness differ by only 2.2 %. Although the comparison shows that deviations increase with thickness, as analyzed and explained in the following text, it still indicates that the technique remains viable even with a sample thickness of 2 mm. Therefore, compared with the laser-flash technique, which is suitable for measuring film-like materials, this method has a broader testing range.

### 4.2. Analysis of Experimental Results of Graphite Bipolar Plate Products

**Figure 11:**
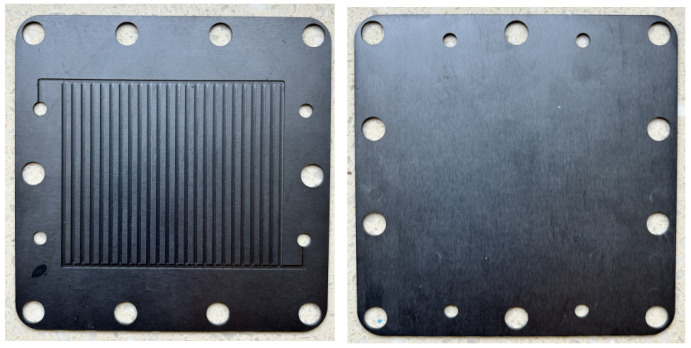
Photographic of graphite bipolar plate sample.

**Figure 12:**
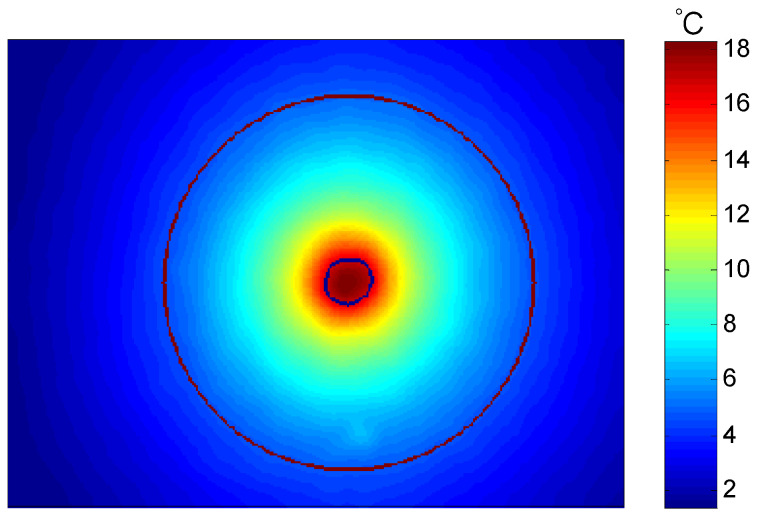
Infrared thermal image of graphite bipolar plate.

Based on the accuracy and feasibility of the aluminum materials testing experiment mentioned above, further testing experiments on graphite bipolar plate products can be conducted. The three tested samples were graphite bipolar plate products used in fuel cells, with dimensions of 100 mm × 100 mm × 0.5 mm, 100 mm × 100 mm × 1 mm, and 100 mm × 100 mm × 1.5 mm, respectively, as shown in Figure 11. Under the same conditions as the aluminum material experiment mentioned above, the average values of the in-plane thermal conductivity for the graphite bipolar plate products with the three different thicknesses were obtained from ten independent measurements. The infrared thermal image of the graphite bipolar plate products is shown in Figure 12, and the results are listed in Table 8, Table 9 and Table 10. Similarly, the absolute and relative standard deviations for ten measurements were calculated and considered as type A uncertainty and reported in Table 8, Table 9 and Table 10. The repeatability resulted within 3%. The experimental results of the 1 mm thick graphite bipolar plate show a deviation of 4.7% from the reference value of the graphite simulation model mentioned above.

A comparison of test results for graphite bipolar plates of three different thicknesses, as presented in Table 8, Table 9 and Table 10, reveals that the average in-plane thermal conductivity rises with increasing sample thickness. This trend is likely associated with variations in through-plane and in-plane heat conduction, along with temperature gradients. Specifically, a thicker sample enhances through-plane heat conduction and alters the temperature gradient along the in-plane direction, which in turn impacts the measured values. However, the relative standard deviation of all test results for samples with three thicknesses was calculated comprehensively, and the repeatability was 2.6%, indicating that this measurement method exhibits high consistency and repeatability across samples of different thicknesses.

A number of factors contribute to the combined measurement uncertainty (type B) associated with the method, which can be classified into the following categories: discrepancies between actual and theoretical models, approximations assumed in the approach, and uncertainties in the measurement of the input parameters, such as sample size, infrared temperature, etc. Among them, the amount of data to be processed affected by temperature measurement noise is the main error in the estimation of in-plane thermal conductivity, especially the estimation of temperature data for a series of sampling points on the testing loop. For thicker samples, due to the temperature gradient in the through-plane direction and relatively low SNR, these data are more susceptible to temperature measurement errors. Additionally, the thicker the sample, the more sensitive it is to measurement noise.

## 5. Conclusions

This study presents a novel nondestructive measurement method based on infrared thermal imaging technology for rapidly detecting the in-plane thermal conductivity of bipolar plates in fuel cells. This method addresses the limitations of traditional methods that have strict demands for the thermal excitation, boundary, and size of materials. The plausibility was confirmed through finite element simulation, while experimental measurements of aluminum materials and graphite bipolar plate products validated the viability of the method. This method can be applied to the thermal management of PEM fuel cells to improve the reaction effectiveness, durability, and general performance. Therefore, it is anticipated that the heat flow loop integration method discussed in this paper, along with the available results of in-plane thermal conductivity of graphite bipolar plates, will contribute to the knowledge repository regarding PEM fuel cells. Furthermore, it will furnish dependable benchmark data for modelers to predict the temperature distribution within a fuel cell precisely. This aspect holds significant importance in the design and thermal management of PEM fuel cells and stacks.

## Figures and Tables

**Table 1:** Thermophysical parameters of the model.

Description	Graphite Material	Graphite Bipolar Plate
In-thermal conductivity	330 W/(m·K)	
Thermal diffusivity	2.1 × 10^−4^ m^2^/s	
Density	2026 kg/m^3^	
Specific heat	775 J/(kg·K)	

**Table 2:** Comparison of simulation results.

Material	Simulation Value W/(m °C)	Reference Value W/(m·°C)	Deviation %
Graphite material	335.8	330.0	1.8
Graphite bipolar plate	334.8	330.0	1.5

**Table 3:** Parameter table of the laser.

Parameter	Data
Power stability	5%
Center wavelength	915 ± 10 nm
Beam divergence angle	440 mrad
Beam diameter of light outlet	5 mm ± 1 mm

**Table 4:** Parameters of the infrared thermal imager (MAGNITY MAG32).

Parameter	Data
FOV (Angle of view)/Focal length	15° × 11.5°/25 mm
Spatial resolution (IFOV)	0.68 mrad
Detector pixel spacing	17 μm
Measuring range	−20 to 150 °C
Thermal sensitivity	<60 mK

**Table 5:** Experimental results of the 6061 aluminum material with a 1 mm thickness.

Group	Measured Value of Thermal Conductivity/W(m·K)^−1^
1	176.12
2	168.37
3	165.64
4	166.97
5	171.30
6	172.64
7	170.04
8	169.00
9	168.71
10	166.68
Average value	169.55
Deviation	0.3%
Standard deviation	3.14
Relative standard deviation	1.9%

**Table 6:** Experimental results of the 1060 aluminum material with a 1 mm thickness.

Group	Measured Value of Thermal Conductivity/W(m·K)^−1^
1	226.43
2	227.27
3	217.43
4	228.00
5	224.17
6	218.93
7	227.21
8	235.67
9	229.19
10	224.58
Average value	225.89
Deviation	4.3%
Standard deviation	5.16
Relative standard deviation	2.3%

**Table 7:** Experimental results of the 1060 aluminum material with a 2 mm thickness.

Group	Measured Value of Thermal Conductivity/W(m·K)^−1^
1	240.05
2	222.52
3	226.18
4	235.20
5	233.95
6	237.24
7	236.05
8	227.02
9	223.89
10	227.65
Average value	230.98
Deviation	2.1%
Standard deviation	6.20
Relative standard deviation	2.7%

**Table 8:** Experimental results of graphite bipolar plate with 0.5 mm thickness.

Group	Measured Value of Thermal Conductivity/W(m·K)^−1^
1	309.39
2	312.68
3	305.03
4	313.79
5	313.99
6	312.18
7	313.91
8	313.36
9	311.45
10	314.02
Average value	311.98
Standard deviation	2.85
Relative standard deviation	0.9%

**Table 9:** Experimental results of graphite bipolar plate with a 1 mm thickness.

Group	Measured Value of Thermal Conductivity/W(m·K)^−1^
1	327.11
2	323.72
3	310.07
4	303.42
5	306.53
6	301.43
7	317.26
8	311.09
9	316.23
10	327.25
Average value	314.41
Standard deviation	9.46
Relative standard deviation	3.0%

**Table 10:** Experimental results of graphite bipolar plate with a 1.5 mm thickness.

Group	Measured Value of Thermal Conductivity/W(m·K)^−1^
1	327.63
2	323.74
3	316.77
4	330.06
5	321.42
6	329.40
7	318.09
8	314.39
9	333.42
10	319.88
Average value	323.48
Standard deviation	6.40
Relative standard deviation	2.0%

## Data Availability

Restrictions apply to the availability of these data. The datasets presented in this article are not readily available because of company restrictions. Requests to access the datasets should be directed to the corresponding author.
